# The Association Between Informal Caregiving and Poor Self-rated Health Among Ever-married Women in Japan: A Nationally Representative Survey

**DOI:** 10.2188/jea.JE20200320

**Published:** 2022-04-05

**Authors:** Yuka Suzuki, Kaori Honjo

**Affiliations:** Social and Behavioral Sciences, Faculty of Medicine, Osaka Medical and Pharmaceutical University, Osaka, Japan

**Keywords:** nursing care, childcare, dual care, self-rated health, sandwich generation

## Abstract

**Background:**

The number of people providing informal caregiving, including dual care, which is the combination of child and nursing care, is increasing. Due to the burden of multiple responsibility, dual care could negatively affect the health of informal caregivers. Previous research has not studied the effects of combining different types of informal caregiving. Therefore, we examined, among Japanese women, 1) the association between types of informal caregiving and self-rated health (SRH), and 2) difference in this association according to caregivers’ socio-economic conditions.

**Methods:**

We analyzed the nationally representative 2013 Comprehensive Survey of Living Conditions data of 104,171 women aged 20–59 years. The odds ratios (ORs) for poor SRH by type of informal caregiving (no care, childcare, nursing care, and dual care) were estimated using logistic regression. We also conducted sub-group analyses by socio-economic conditions (equivalent monthly household expenditure and educational attainment).

**Results:**

Compared to the no care group, the adjusted ORs for poor SRH of the childcare, nursing-care, and dual care groups were 0.92 (95% confidence interval [CI], 0.88–0.97), 1.33 (95% CI, 1.21–1.47), and 1.42 (95% CI, 1.23–1.64), respectively. There was no extra risk arisen from combining childcare and nursing care. The sub-group analyses indicated that neither household expenditure nor educational attainment affected the association between caregiving type and poor SRH.

**Conclusion:**

Our study found that informal nursing care and dual care impose a health burden on female caregivers, regardless of their socio-economic conditions. This highlights the importance of addressing the effects of informal caregiving on the health of women.

## INTRODUCTION

Recently, it has been observed that in many countries, the age at which people have children, at which they become independent from their parents, and the age of societies as a whole have increased. This demographic trend has resulted in an increased number of people who informally care both for their children and other family members needing support due to illness or decline in physical function. These individuals are called dual caregivers in a “sandwich generation.”^1^ Many of these dual caregivers feel that they are pulled in many directions. They struggle to manage the competing demands of their dependent children and other family members needing care, as well as of other aspects of their own lives, including their jobs. Dual caregivers are typically in their 40s to 50s, at the peak of their career, a situation that imposes additional demands.^2^ These conflicting personal demands may cause stress and detrimental health effects.^3^

Globally, the population of people aged 60 years or over is projected to grow faster than any other age group by 2050, and middle-income countries shall be no exception.^4^ As a result, a greater proportion of people of working age would be supporting their family members. The health of dual caregivers would become a matter of concern in the future. Thus, capturing the current status through epidemiological studies is important to improve the health of these caregivers.

Several studies have assessed the relationship between informal childcare or nursing care and the health of caregivers. Reports have shown the detrimental health effects of informal childcare, especially on women who work full-time,^5^^,^^6^ as parenting requires time and energy and includes many demands.^6^ Nevertheless, the results of various studies are not conclusive.^7^ There is clear evidence of the negative effects of informal nursing care on caregivers’ self-rated health (SRH), as demonstrated in a recent longitudinal study conducted in Germany,^8^ as well as in a nationwide cross-sectional study in Sweden.^9^ Informal nursing care is a source of chronic stress.^10^ Moreover, caregivers who live with care recipients report worse SRH than do non-caregivers.^11^

Previous studies have primarily focused on the association between informal childcare or nursing care and health. The health effects of dual care on caregivers have yet to be thoroughly examined. Recently, organizations in the United Kingdom and the United States investigated and described the health concerns among dual caregivers.^12^^,^^13^ However, epidemiological research remains scarce. Do et al^14^ conducted one of the few studies that focused on the health effects of dual care in the United States. They analyzed nationwide survey data and reported that people who provided both informal childcare and nursing care had higher risk of poor SRH compared to those who provided only informal nursing care.

Chisholm^3^ noted that informal caregiving is influenced by socio-cultural factors, as each country has its own social norms and culture related to the family system. That is, the sense of obligation or burden that caregivers feel depends on how the society views informal caregiving. Some cultures consider caring for an elderly parent as an honor. In those instances, caregivers might consider informal nursing care to be less burdensome; other cultures may exhibit a different perspectives.^3^ Thus, studies of the health effects of informal caregiving, including dual care, should be conducted considering its specific cultural context.

In Japan, the age of giving birth to the first child increased by 5 years over a 30-year period, from 25.7 years in 1975 to 30.7 years in 2015,^15^ due to diverse socio-economic factors. These factors included an increase in the level of academic achievement among women and female participation in the labor market.^16^ The proportion of population aged 65 years or older was 28.1% in 2018, and is projected to continue to increase.^17^ Combined with the increase in the age of giving birth, the higher proportion of mothers are expected to provide informal nursing care for their parents in the near future.

Japanese society has relied on nursing care provided largely by family members of the care recipients. A recent nationwide survey reported that 58.7% of people in need of support were cared for primarily by their co-habiting family members, whereas only 13.0% received care from long-term care service providers as their primary caregivers.^18^ Hence, family members currently play an important role in providing long-term care in Japan. In addition, traditional gender norms, which consider women to be family caregivers while men work outside the home, leaving the chores and family duties to women, still exists in Japan.^18^^,^^19^ Thus, informal caregiving, including dual care, in Japan is performed predominantly by women. In Japan in 2016, an estimated 253,000 people (168,000 women and 85,000 men) provided dual care.^20^ Along with the continuously increasing proportion of older people, and the consequent increase in the number of women providing dual care, it is important to examine the effect of informal caregiving especially dual care on women’s health. As women play an essential role in supporting their families,^18^^,^^19^ helping them remain healthy would also help other family members remain healthy.

Socio-economic conditions are a factor that may change the relationship between caregiving and caregivers’ health. One’s socio-economic conditions might define the obtainable amount of housekeeping, babysitting, or care services, and this might affect the SRH of caregivers. Possible acquisition of help among caregivers with substantial financial resources might ease the burden associated with multiple roles added by informal caregiving.^5^ Regarding nursing care, Do et al^14^ reported that dual caregivers in lower-income groups potentially exhibited poorer SRH than did those in a higher-income group. However, the results were not conclusive and depended on the number of children and race/ethnicity of the participants. Thus, the socio-economic conditions of informal caregivers must be considered when studying the relationship between informal caregiving and caregivers’ health.

We believe studying health effects of informal caregiving, especially dual care, would nurture scientific grounds for public health policymaking to help support women needed both inside and outside of their house in this ageing society. Accordingly, we examined, using a nationally representative survey data of Japanese women, 1) the association between the types of informal caregiving (no care, childcare only, nursing care only, and dual care) and SRH, and 2) the difference in the relationship according to the caregivers’ socio-economic conditions.

## METHOD

### Study population

We analyzed the nationwide, self-administered questionnaire data of the 2013 Comprehensive Survey of Living Conditions (CSLC). The CSLC is a triannual, nationally representative survey that is conducted by the Ministry of Health, Labour and Welfare (MHLW). The questionnaires for the 2013 survey were distributed to 295,367 households that were randomly selected, of which 234,383 (79.4%) responded with valid answers.^21^ The data from the CSLC2013 were obtained from the MHLW for this analysis, according to the requirements of the Statistics Act, Article 33. Of the 106,779 ever-married female respondents aged 20–59 years, we excluded 2,608 women whose SRH data were missing. The data from the remaining 104,171 women (our final study population) were included in the analysis.

Ethics approval was not necessary, as CSLC was conducted by MHLW under the Statistics Act, and our data offered by MHLW did not contain any personally identifiable information.

### Measurement

#### Independent variable

Our independent variable was the type of informal caregiving. Women were categorized into four groups according to their care roles: 1) no care, 2) childcare only, 3) nursing care only, and 4) dual care (both childcare and nursing care). Childcare was defined as a woman living with her under-18-year-old child. Nursing care was defined as a woman who was the main caregiver for her family member aged 6 years or older who needed support due to illness or decline in physical function.

#### Dependent variable

Our dependent variable was poor SRH, which was an established measurement of general health status of the respondent that well predicts morbidity and mortality.^22^ In this study, SRH was assessed using the question “How is your current health status?” with five possible responses, “excellent,” “very good,” “good,” “not so good,” or “not good.” Poor SRH was defined as the responses “not so good,” or “not good.”

#### Covariates

The hypothesized effect modifier was socio-economic conditions, which were assessed as equivalent monthly household expenditure and educational attainment. The equivalent monthly household expenditure^23^ was calculated by dividing household expenditure in May of the survey year by the square root of the number of household members, and categorized into two groups (low/high; mean equivalent expenditure, 89,482 yen and 204,480 yen, respectively). Educational level was measured by the individual’s highest educational attainment, categorized as low (junior high school or less, high school) and high (junior college, university and higher).

Possible confounding variables used for statistical adjustment consisted of 5-year age groups, educational attainment (junior high school or less, high school, junior college, university and higher), marital status (currently married, or not), job class of the highest wage earner in the household (professional or managerial, white collar, blue collar, other, or not working), equivalent monthly household expenditure (quartiles), and prefecture groups.

### Statistical analysis

Basic characteristics by type of informal caregiving were compared using the chi-square test. The odds ratios (ORs) for poor SRH by type of informal caregiving were estimated using multivariable logistic regression. The significance of the interaction terms between childcare and nursing care were estimated to determine whether excess risk was associated with providing both childcare and nursing care. We estimated the ORs for each caregiving type with reference to the no care group, adjusting for possible confounding factors.

We also conducted a sub-group analysis according to the respondents’ equivalent monthly household expenditure in halves (low/high), as well as educational attainment. Each of the four caregiving types (no care, childcare, nursing care, dual care) were divided by expenditure groups (low/high), yielding eight groups in total. The ORs for poor SRH were estimated with reference to the no care category in the high-expenditure group. A similar analysis was conducted according to educational attainment. The statistical software used was SAS 9.4 (SAS Institute, Cary, NC, USA).

## RESULTS

Table 1 shows the basic characteristics of the study population according to the type of informal caregiving provided. In total, 11.9% of the study population reported poor SRH. Most of the study population was in their 40s and 50s (68.3%). Women in the childcare group were the youngest and the women in the nursing-care group were the oldest. The mean ages of those in the no care group, childcare group, nursing-care group, and dual care group were 49.8, 39.4, 53.8, and 43.3 years, respectively. Most (88.0%) were currently married. Of the study population, 44.4% were in the high-education group. Among all caregiving types, the high-education group was found the most in the childcare group (49.8%) and the least in the nursing-care group (36.8%). Women in the no care group were most likely to be in the high monthly spending category (55.9%), whereas women in the dual care group were least likely to be in that category (40.3%).

**Table 1.** Characteristics of ever-married women aged 20–59 years, according to the types of informal caregiving

			Types of informal caregiving								*P* for difference
	
	All		No care		Childcare		Nursing care		Dual care		
	104,171		46,483		53,215		2,985		1,488		
				
	*n*	%	*n*	%	*n*	%	*n*	%	*n*	%	
**Poor self-rated health**											
Yes	12,375	11.9	6,086	13.1	5,512	10.4	529	17.7	248	16.7	<0.001
**Age, years**											
	44.5		49.8		39.4		53.8		43.3		<0.001
**Age group, years**											<0.001
20–24	1,112	1.1	393	0.9	714	1.3	0	0.0	5	0.3	
25–29	5,384	5.2	1,661	3.6	3,684	6.9	13	0.4	26	1.8	
30–34	10,783	10.4	2,129	4.6	8,536	16.0	15	0.5	103	6.9	
35–39	15,763	15.1	2,231	4.8	13,234	24.9	38	1.3	260	17.5	
40–44	17,975	17.3	3,149	6.8	14,314	26.9	75	2.5	437	29.4	
45–49	16,910	16.2	6,816	14.7	9,351	17.6	323	10.8	420	28.2	
50–54	17,395	16.7	13,346	28.7	2,944	5.5	908	30.4	197	13.2	
55–59	18,849	18.1	16,758	36.1	548	0.8	1,613	54.0	40	2.7	
**Educational attainment**											<0.001
Junior high school, or less	4,282	4.1	2,315	5.0	1,741	3.3	172	5.8	54	3.6	
High school	43,596	41.9	21,326	45.9	20,168	37.9	1,453	48.7	649	43.6	
Junior college	32,314	31.0	12,563	27.0	18,405	34.6	853	28.6	493	33.1	
University and higher	13,935	13.4	5,449	11.7	8,075	15.2	245	8.2	166	11.2	
Missing	10,044	9.6	4,830	10.4	4,826	9.1	262	8.8	126	8.5	
**Educational attainment level**											
High	46,249	44.4	18,012	38.8	26,480	49.8	1,098	36.8	659	44.3	<0.001
**Married**											
Yes	91,690	88.0	39,669	85.3	48,214	90.6	2,496	83.6	1,311	88.1	<0.001
**Job category of the highest wage earner in the household**											<0.001
Professional, managerial job	36,224	34.8	15,807	34.0	19,033	35.8	902	30.2	482	32.4	
White collar job	29,050	27.9	12,876	27.7	15,078	28.3	686	23.0	410	27.6	
Blue collar job	25,476	24.5	11,246	24.2	13,116	24.7	749	25.1	365	24.5	
Others	6,503	6.2	2,743	5.9	3,461	6.5	196	6.6	103	6.9	
Not working	5,460	5.2	3,123	6.7	1,803	3.4	424	14.2	110	7.4	
Missing	1,458	1.4	688	1.5	724	1.4	28	0.9	18	1.2	
**Equivalent monthly household expenditure, quartiles**											<0.001
Q1 (Low)	25,142	24.1	8,802	18.9	15,203	28.6	706	23.7	431	29.0	
Q2	25,935	24.9	9,624	20.7	15,217	28.6	712	23.9	382	25.7	
Q3	23,391	22.5	10,742	23.1	11,625	21.9	669	22.4	355	23.9	
Q4 (High)	25,319	24.3	15,255	32.8	9,023	27.0	796	26.7	245	16.5	
Missing	4,384	4.2	2,060	4.4	2,147	4.0	102	3.4	75	5.0	
**Expenditure level**											
High	48,710	46.8	25,997	55.9	20,648	48.8	1,465	49.1	600	40.3	<0.001

Table 2 shows that there was a significant association between the type of informal caregiving and SRH. Compared to the no care group, the adjusted ORs for poor SRH of women in the childcare, nursing care, and dual care groups were 0.92 (95% CI, 0.88–0.97), 1.33 (95% CI, 1.21–1.47), and 1.42 (95% CI, 1.23–1.64), respectively. The *P*-value for the interaction term between childcare and nursing care was 0.10, indicating there was no extra risk arisen from combining childcare and nursing care.

**Table 2.** Odds ratios for self-rated poor health associated with the types of informal caregiving among ever-married Japanese women aged 20–59 years

	Type of informal caregiving							*P* for interaction between childcare and nursing care
	
*n*	No care	Childcare		Nursing care		Dual care		
	46,483	53,215		2,985		1,488		
			
	OR	OR	95% CI	OR	95% CI	OR	95% CI	
n cases	6,086	5,512		529		248		0.10
Crude	1.00	0.77	(0.74, 0.80)	1.43	(1.30, 1.58)	1.33	(1.16, 1.53)	
Adjusted^*^	1.00	0.92	(0.88, 0.97)	1.33	(1.21, 1.47)	1.42	(1.23, 1.64)	

CI, confidence interval; OR, odds ratio.

^*^Adjusted for age group, educational attainment, job class of the highest wage earner in the household, marital status (married, or not), equivalent monthly household expenditure, and prefecture groups.

Table 3 shows the results of the sub-group analysis by the equivalent monthly household expenditure and educational attainment. The sub-group analysis by expenditure groups showed that ORs of no care groups did not significantly differ between expenditure groups. The adjusted OR for no care individuals in the low-expenditure group was 0.99 (95% CI, 0.94–1.05) compared to no care individuals in the high-expenditure group. Childcare groups had slightly lower ORs in both high- and low-expenditure groups, compared to no care individuals in the high-expenditure group: 0.96 (95% CI, 0.90–1.03) and 0.88 (95% CI, 0.83–0.94), respectively. In contrast, nursing-care and dual care groups had significantly higher ORs in both expenditure groups. The respective adjusted ORs for nursing care and dual care groups compared to the no care with the high-expenditure group were 1.31 (95% CI, 1.14–1.51) and 1.55 (95% CI, 1.25–1.92) in the high-expenditure group, and 1.31 (95% CI, 1.14–1.51) and 1.34 (95% CI, 1.10–1.63) in the low-expenditure group. Interactions between expenditure and caregiving types were not significant (data not shown), demonstrating that the association between type of informal caregiving and SRH did not significantly differ by household expenditure level.

**Table 3.** Odds ratios for self-rated poor health associated with the types of informal caregiving by equivalent monthly household expenditure and educational attainment

	Type of informal caregiving							

	No care		Childcare		Nursing care		Dual care	
			
	OR	95% CI	OR	95% CI	OR	95% CI	OR	95% CI
**Household expenditure**								
**High**								
*n*	25,997		20,648		1,465		600	
n cases	3,299		2,235		248		107	
Crude	1.00		0.84	(0.79, 0.88)	1.40	(1.22, 1.62)	1.49	(1.21, 1.85)
Adjusted^*^	1.00		0.96	(0.90, 1.03)	1.31	(1.14, 1.51)	1.55	(1.25, 1.92)
**Low**								
*n*	18,426		30,420		1,418		813	
n cases	2,487		3,038		259		130	
Crude	1.07	(1.02, 1.14)	0.76	(0.72, 0.80)	1.54	(1.34, 1.77)	1.31	(1.08, 1.59)
Adjusted^*^	0.99	(0.94, 1.05)	0.88	(0.83, 0.94)	1.31	(1.14, 1.51)	1.34	(1.10, 1.63)

**Educational attainment**								
**Junior college or higher**								
*n*	18,012		26,480		1,098		659	
n cases	2,108		2,492		179		110	
Crude	1.00		0.78	(0.74, 0.83)	1.47	(1.24, 1.74)	1.51	(1.23, 1.86)
Adjusted^**^	1.00		0.92	(0.86, 0.98)	1.30	(1.10, 1.54)	1.58	(1.28, 1.96)
**High school or lower**								
*n*	23,641		21,909		1,625		703	
n cases	3,295		2,478		294		121	
Crude	1.22	(1.15, 1.30)	0.96	(0.91, 1.02)	1.67	(1.46, 1.91)	1.57	(1.28, 1.92)
Adjusted^**^	1.14	(1.07, 1.21)	1.08	(1.00, 1.15)	1.48	(1.29, 1.69)	1.58	(1.29, 1.94)

CI, confidence interval; OR, odds ratio.

^*^Adjusted for age group, educational attainment, job class of the highest wage earner in the household, marital status (married, or not), and prefecture groups.

^**^Adjusted for age group, job class of the highest wage earner in the household, marital status (married, or not), equivalent household monthly expenditure, and prefecture groups.

The results of sub-group analysis by educational attainment also indicated that the association between caregiving type and poor SRH did not differ by education level. In each education group, ORs of the childcare groups were lower, those of the nursing-care groups were higher, and those of the dual care groups were even higher, as compared to the no care groups. Among the no care groups, the low-education group had a somewhat higher OR than the high-education group. The interaction between educational level and caregiving type were not significant (data not shown).

## DISCUSSION

In this study of nationally representative Japanese ever-married women aged 20–59 years, we identified a significant association between informal caregiving and SRH. Compared to the women who did not provide care, women who only provided childcare were less likely to report poor SRH. In contrast, women who provided nursing care or dual care were more likely to report poor SRH. Women in the dual care group were more likely to exhibit poor SRH than those in the nursing-care group, even though the interaction between childcare and nursing care was not statistically significant. In addition, the identified association did not differ by household expenditure or educational attainment.

Women performing dual care had a higher OR of poor SRH than did non-caregivers. To the best of our knowledge, no previous study has compared the difference in SRH between dual caregivers and non-caregivers. Dual care requires that caregivers expend time and energy to take care of their children and other family members. This high level of responsibility causes role conflicts and stress, and can possibly lead to health-related stress or work-family imbalance.^3^ As a result, dual care might reduce caregivers’ time and motivation for self-care; this is a reasonable assumption, as the less-healthy behaviors of dual caregivers have already been documented.^24^ Allowing women providing informal caregiving unhealthy would affect their family members as well, as women play an essential role in supporting their families.^18^^,^^19^

Our study showed that the risk of poor SRH among dual caregivers did not differ significantly from that of nursing caregivers. Thus, our study is inconsistent with a study conducted in the United States which found that dual caregivers were more likely to have poor SRH than nursing caregivers.^14^ Our study indicated a lesser degree of excess risk among the dual caregivers perhaps because of a protective effect of the task of childcare on SRH, which might have eased the normally detrimental effects of performing dual care.

Women performing nursing care were more likely to report poor SRH than were non-caregivers, consistent with previous studies.^8^^,^^14^ Nursing caregivers are exposed to chronic stress and excessive demands.^9^ This situation possibly led nursing caregivers to have a higher OR of reporting poor SRH than non-caregivers.

Regarding the association between childcare and SRH, we found that women performing childcare had a lower risk of poor SRH than did non-caregivers. Indeed, childcare provides mothers with the opportunity to construct a social network and enhance self-esteem,^5^ which could contribute to mothers’ better subjective health. Further, mothers with small children are known to practice better health behaviors, such as eating breakfast, avoiding harmful alcohol intake, and not smoking, than nationally representative Japanese women in a similar age group.^25^ Another possible explanation for our results is the “healthy mothers effect,” in which healthy women are more likely to have children than less healthy women.^6^

According to our sub-group analysis, socio-economic conditions did not change the relationship between caregiving and SRH. This is contrary to previous studies, which reported socio-economic conditions act as an effect modifier of the association between caregiving and SRH.^6^^,^^14^ Several mechanisms have been discussed regarding how socio-economic conditions alter the relationship between informal caregiving and SRH. A previous study found that women with multiple roles experienced less psychological distress if they had abundant financial resources to alleviate their burden associated with domestic roles.^5^ The indication is that those women could outsource their domestic duties using their financial resources. However, our study found no relationship between socio-economic conditions and the health effects of caregiving. This may have been because the participants with abundant financial resources in our study might not have outsourced their domestic duties. In Japan, housekeeping, childcare, and nursing care are predominantly considered tasks for the family (more precisely women), rather than for outsourcing.^26^ This social norm might have rendered high socio-economic conditions (ie, financial advantages) less likely to reduce women’s burdens. Furthermore, Japan’s unique social welfare system might have filled the social gap. The universal Long-Term Care Insurance System is a public social insurance scheme, introduced by the MHLW in 2002. People aged 40 years or greater pay a monthly premium to their health care insurers. People can receive necessary nursing care services either in their home or at care facilities with a copayment of 10–30% of the service fee. The proportion of copayment and its upper limit depends on the care recipients’ income. The amount of eligible service is determined based on the care recipients’ psychological and physical condition.^27^ Within this universal care insurance system, people can receive care benefits regardless of their income. This access to benefits may have equalized the caregiving burden among informal caregivers of different socio-economic conditions.

Our study is one of few that focused on the health effects of childcare, nursing care, and dual care using nationally representative survey data. As dual caregivers represent a minority of the total survey population (1.4% in our data), a large-scale study was suitable to gain understanding of the general status of these caregivers. We believe our study contributed to scientific evidence on health effects of informal caregiving, especially dual care, on female caregivers in an ageing society.

Nevertheless, some limitations must be noted. First, due to the cross-sectional nature of the study, we could not assess the causality of the results. Future longitudinal studies are therefore required. Second, there is the possibility of selection bias. Those who take care of other family members would have been generally healthy. Third, there may be measurement error that might have underestimated the results. We defined a woman providing childcare as an individual living with her 18-year-old or younger children. Living with children might not always represent being engaged in childcare. Also, we defined nursing care as informal caregiving for a person aged 6 or over. There might be some overlapping care role between childcare and nursing care if the nursing care recipient was a child. Further, we measured socio-economic conditions using household expenditure rather than income, and by the women’s own educational level. These criteria may not precisely indicate the women’s socio-economic status. Finally, there may be residual confounding, although we adjusted our analyses to take various plausible confounding factors into account.

### Conclusion

Our study found that informal nursing care and dual care impose a health burden on female caregivers, regardless of their socio-economic conditions. This highlights the importance of addressing the health of women who provide care for their family members on an informal basis.
